# miRNA Sequencing Analysis in Maize Roots Treated with Neutral and Alkaline Salts

**DOI:** 10.3390/cimb46080524

**Published:** 2024-08-15

**Authors:** Ziqi Chen, Yang Liu, Qi Wang, Jianbo Fei, Xiangguo Liu, Chuang Zhang, Yuejia Yin

**Affiliations:** Institute of Agricultural Biotechnology/Jilin Provincial Key Laboratory of Agricultural Biotechnology, Jilin Academy of Agricultural Sciences (Northeast Agricultural Research Center of China), Changchun 130033, China; chenziqi@cjaas.com (Z.C.); liuyang_20045894@163.com (Y.L.); wang-edu@foxmail.com (Q.W.); 13041382812@163.com (J.F.); lxgyyj@cjaas.com (X.L.)

**Keywords:** miRNA sequencing analysis, maize root, neutral salt, alkaline salts

## Abstract

Soil salinization/alkalization is a complex environmental factor that includes not only neutral salt NaCl but also other components like Na_2_CO_3_. miRNAs, as small molecules that regulate gene expression post-transcriptionally, are involved in plant responses to abiotic stress. In this study, maize seedling roots were treated for 5 h with 100 mM NaCl, 50 mM Na_2_CO_3_, and H_2_O, respectively. Sequencing analysis of differentially expressed miRNAs under these conditions revealed that the Na_2_CO_3_ treatment group had the most differentially expressed miRNAs. Cluster analysis indicated their main involvement in the regulation of ion transport, binding, metabolism, and phenylpropanoid and flavonoid biosynthesis pathways. The unique differentially expressed miRNAs in the NaCl treatment group were related to the sulfur metabolism pathway. This indicates a significant difference in the response patterns of maize to different treatment groups. This study provides theoretical evidence and genetic resources for further analysis of the molecular mechanisms behind maize’s salt–alkali tolerance.

## 1. Introduction

Saline–alkali soils arise due to the progressive accumulation of salts within the soil matrix, detrimentally impacting agricultural productivity. Data from UNESCO and the Food and Agriculture Organization indicate that globally, saline–alkali soils encompass approximately 954 million hectares, with China accounting for 99.13 million hectares of this total [1]. In China, the genesis of such soils is predominantly linked to the accretion of carbonates, which elevates the soil’s alkalinity to levels that are inhospitable for plant life, particularly in regions where salinity is acute. Given that saline–alkaline soils contain both neutral and basic salts, requiring plants to cope with various ionic stresses and pH changes, studying the comprehensive effects of salt and alkali on plants is of great significance for understanding the physiological response mechanisms of plants in saline–alkaline environments and for improving the salt–alkali tolerance of crops.

Unlike NaCl stress, Na_2_CO_3_ stress in plants arises from a complex interaction of sodium ion stress, high pH stress, and carbonate stress, with intricate mechanisms of action [2]. Previous studies on salinity–alkalinity stress focused mainly on neutral salts such as NaCl, identifying osmotic stress, ionic stress, and oxidative stress as the primary effects on plants [3]. Additionally, Na_2_CO_3_ stress incorporates a high pH effect, which is known to directly interfere with nutrient absorption, organic acid balance, and ion equilibrium, especially impacting pH stability at the cellular and whole-plant levels [4]. Owing to its triple stress effects of Na^+^, high pH, and CO_3_^2^^−^, Na_2_CO_3_ poses greater risks to plants. The effects of Na⁺ and CO_3_^2^⁻ stress on plants are primarily due to their toxic effects and the resulting nutritional imbalance. High concentrations of Na⁺ disrupt the ionic balance within cells, replacing potassium ions (K⁺) and interfering with normal physiological functions, including enzyme activity and water balance [5]. Furthermore, the accumulation of Na⁺ increases the osmotic pressure of the soil solution, making it difficult for plant roots to absorb water, thus leading to water stress [6]. On the other hand, CO_3_^2^⁻ raises the pH of the soil, making it alkaline. High pH levels can lead to a decrease in the solubility of many essential elements (such as iron, manganese, and zinc), making it difficult for plants to absorb these necessary micronutrients [7]. Additionally, high concentrations of carbonate ions can interfere with plant metabolic activities, affecting normal root respiration and nutrient uptake. Therefore, Na⁺ and CO_3_^2^⁻ stress primarily affects plants through ionic toxicity and nutritional imbalance, leading to inhibited growth and impaired physiological functions. Research by Parida and Das [8] indicates that Na_2_CO_3_ stress disrupts the dynamic balance of reactive oxygen species, causing oxidative damage to enzymes and photosynthetic apparatus, thereby suppressing the energy expenditure for CO_2_ absorption. Badger and Price [9] found that Na_2_CO_3_ stress inhibits the activity of key enzymes in the Calvin cycle, reducing carbon fixation efficiency and photosynthesis. Miller et al. [10] observed enhanced lipid peroxidation under Na_2_CO_3_ stress, leading to significant electrolyte leakage and malondialdehyde (MDA) content, suggesting that the plasma membrane is the initial site of damage. Studies have shown that the concentration of Na^+^ and K^+^ on the surface of *Puccinellia tenuiflora* leaves increases with Na_2_CO_3_ concentration, as the plant expels salt ions through stomatal openings or excretes waxy substances, thereby achieving salinity–alkalinity tolerance [11,12]. Furthermore, Puccinellia tenuiflora’s adaptive regulatory mechanism to high pH stress caused by alkalinity suggests that the plant adjusts the pH primarily through the accumulation and secretion of organic acids, such as citric acid, from the roots. Under NaHCO_3_ stress, an increase in citrate content in leaves and roots was observed, along with the induction of vacuolar Na^+^/H^+^ antiporters, indicating their crucial role in pH regulation [13,14]. These studies indicate that Na_2_CO_3_ stress triggers a series of physiological and molecular changes in halophytes to combat alkaline salt stress. However, glycophytes like maize might have distinct mechanisms in responding to Na_2_CO_3_ stress compared to halophytes.

Plant stress responses involve complex and precise regulatory processes, and miRNAs, as a class of small RNAs crucial for post-transcriptional regulation, play an extremely important role in plant growth, development, and response to abiotic and biotic stresses by directly targeting functional genes or indirectly regulating downstream functional genes through targeting transcription factors. Over the years, with the widespread application of advanced high-throughput sequencing technologies, studies have reported that miRNAs in plants exhibit high conservation and play roles in plant growth and development, various treatment responses, and are considered important regulators in the post-transcriptional regulation of plant salt stress responses [15,16]. Xu et al. [17] propose future directions for the study of plant miRNAs under salt stress, suggesting that manipulating microRNAs could enhance the salinity resistance of crops. Chen et al. [18] employed a combination of PacBio isoform sequencing (Iso-Seq) and BGISEQ short-read RNA-seq to investigate the role of miRNAs in the salt stress response of the mangrove plant, Buch.-Ham. Through KEGG analysis, eighteen miRNA targets were associated with ‘environmental information processing’ and linked to plant hormone signal transduction (ko04075), the MAPK signaling pathway-plant (ko04016), and ABC transporters (ko02010).

Currently, most studies on miRNA responses to salt stress focus on neutral salts, while research on alkaline salts is relatively scarce, and the relevant molecular mechanisms remain unclear. This study aims to reveal the expression patterns of miRNAs under different salt stress conditions by comparing the differentially expressed miRNAs in maize roots under neutral salt and alkaline salt conditions. We will employ high-throughput sequencing technology to systematically analyze the miRNA expression profiles in maize roots under neutral and alkaline salt conditions, identifying significantly differentially expressed miRNAs. Furthermore, we will investigate the target genes and regulatory networks of these differentially expressed miRNAs under salt stress conditions to elucidate their mechanisms of action in plant salt stress responses. Additionally, this research aims to provide new insights into the post-transcriptional regulatory mechanisms of plants under salt stress and to offer theoretical support for enhancing crop resilience in saline–alkaline soils, facilitating the breeding of salt–alkaline tolerant crop varieties. By achieving these objectives, we hope to deepen our understanding of the miRNA regulatory mechanisms in maize under neutral and alkaline salt stress, thereby providing scientific evidence and technical support for improving crop growth and tolerance in saline–alkaline soils.

## 2. Materials and Methods

### 2.1. Maize Seedling Stress Treatment

B73 maize seeds were dark-incubated at 28 °C for 3–4 days, then transplanted to a greenhouse (25 °C, 16 h light/8 h dark) for further growth. Hydroponic culture was conducted using a 1/4 strength MS liquid medium. After 8–10 days of greenhouse cultivation, uniformly developed three-leaf stage maize plants were selected. The roots were treated with 100 mM NaCl, 50 mM Na_2_CO_3_, and H_2_O for 5 h. Root tissues from each stress treatment were collected, flash-frozen in liquid nitrogen, and stored at −80 °C for subsequent analysis.

### 2.2. miRNA Sequencing Analysis

Fifteen samples from each group were randomly selected and divided into three sets, constituting three biological replicates for an initial comprehensive miRNA sequencing. The library construction and sequencing work was entrusted to LC Bio Technology Co., Ltd. (Hangzhou, China). The sequencing and analysis methods can be briefly described as follows.

The raw sequencing reads were processed using the in-house software ACGT101-miR (v4.2) to eliminate adapter dimers, low complexity sequences, common RNA families (rRNA, tRNA, snRNA, snoRNA), and repeats. Following this, unique sequences ranging from 18 to 25 nucleotides were mapped to species-specific precursors in miRBase 22.1 via a BLAST search to identify known miRNAs and novel 3p- and 5p-derived miRNAs. Length variations at both the 3′ and 5′ ends, as well as one internal mismatch, were permitted during alignment. Unique sequences mapping to mature miRNAs in the hairpin arms of specific species were classified as known miRNAs. Sequences aligning to the opposite arm of known species-specific precursor hairpins, relative to the annotated mature miRNA-containing arm, were designated as novel 5p- or 3p-derived miRNA candidates. Remaining sequences were mapped to other selected species’ precursors (excluding the specific species) in miRBase 22.1 via BLAST, and the mapped pre-miRNAs were subsequently BLASTed against the specific species’ genomes to determine their genomic locations. These sequences were defined as known miRNAs. Unmapped sequences were BLASTed against the specific genomes, and hairpin RNA structures containing these sequences were predicted from the flanking 120 nucleotide sequences using RNAfold software (http://rna.tbi.univie.ac.at/cgi-bin/RNAWebSuite/RNAfold.cgi, accessed on 13 August 2024. Vienna, Austria). The criteria for secondary structure prediction included the following: (1) number of nucleotides in one bulge in the stem (≤12), (2) number of base pairs in the stem region of the predicted hairpin (≥16), (3) free energy cutoff (kcal/mol ≤−15), (4) length of hairpin (up and down stems plus terminal loop ≥ 50), (5) length of hairpin loop (≤200), (6) number of nucleotides in one bulge in the mature region (≤4), (7) number of biased errors in one bulge in the mature region (≤2), (8) number of biased bulges in the mature region (≤2), (9) number of errors in the mature region (≤4), (10) number of base pairs in the mature region of the predicted hairpin (≥12), and (11) percentage of the mature sequence in the stem (≥80). The differential expression of miRNAs, based on normalized deep sequencing counts, was analyzed using ANOVA. Significance thresholds were set at 0.01 and 0.05 for each test.

The raw date of sequencing file could be accessible with the following link: https://www.ncbi.nlm.nih.gov/sra/PRJNA1126005 (accessed on 1 July 2024).

### 2.3. RNA Extraction, Quantification and Quantitative Real Time PCR (qPCR)

Total RNA was extracted from tissue samples utilizing the EasyPure^®^ miRNA Kit (TransGen, Beijing, China), adhering strictly to the guidelines provided by the manufacturer. To assess the concentration and integrity of the extracted RNA, a Bioanalyzer 2100 instrument from Agilent Technologies (Santa Clara, CA, USA) was employed. Following the evaluation, cDNA synthesis was carried out using the TransScript^®^ One-Step gDNA Removal (TransGen, Beijing, China) and cDNA Synthesis SuperMix (TransGen, Beijing, China) in accordance with the manufacturer’s protocol. The resulting cDNA served as a template for PCR amplification, utilizing gene-specific primer pairs as listed in Table S12. Primers designated for selected miRNA and U6 detection were sourced from Sangon Biotech (Shanghai) Co., Ltd. (Shanghai, China). Quantitative real-time PCR (qPCR) analyses were conducted using the ABI StepOne system (Applied Biosystems Inc., Norwalk, CT, USA) employing the TransStart^®^ Tip Green qPCR SuperMix (+Dye I) kit (TransGen, Beijing, China) for the reactions. Within this experimental setup, U6 RNA was implemented as the endogenous reference for normalizing miRNA expression levels. The expression fold changes in the miRNAs were quantified employing the ΔΔCt method. For the calculation of the relative expression levels of these genes, the 2^−ΔΔCT^ method was utilized, with normalization against U6. This approach facilitated a precise quantification of miRNA expression alterations within the tissue samples under investigation [19].

### 2.4. Computational Prediction of miRNA Targets

To elucidate the possible molecular roles of the differentially expressed candidate miRNAs, two computational target prediction tools, TargetScan and miranda, were employed to identify potential miRNA binding sites. These algorithms were selected for their robust capabilities in predicting miRNA–mRNA interactions based on sequence complementarity and other structural features. The predictions generated by both TargetScan and miranda were subsequently integrated to enhance the reliability of the results. Overlapping predictions between the two algorithms were calculated to identify consensus targets, which are more likely to represent true miRNA–target interactions due to the agreement of independent computational models. Furthermore, the functional annotation of these abundant miRNAs and their predicted targets was conducted using in-house Perl scripts. These scripts were utilized to annotate the Gene Ontology (GO) terms and Kyoto Encyclopedia of Genes and Genomes (KEGG) pathways associated with the miRNA targets.

### 2.5. Ion Content Determination

Sample preparation: A volume of 200 μL of the sample was transferred into a 15 mL polypropylene tube with a screw cap, followed by the addition of 3.8 mL of diluent solution and subsequent vortexing [20].

Determination of sulfate in plants was conducted referencing the paper by Kurmanbayeva A et al. [21].

The methodology for assessing sodium (Na^+^) and potassium (K^+^) concentrations was delineated by Xu et al. [22].

Determination of other ion contents referenced the paper by AbdElgawad H et al. [23].

## 3. Results

### 3.1. Phenotypic Identification of Maize Roots under Neutral Salt and Alkaline Stress

**Figure 1:**
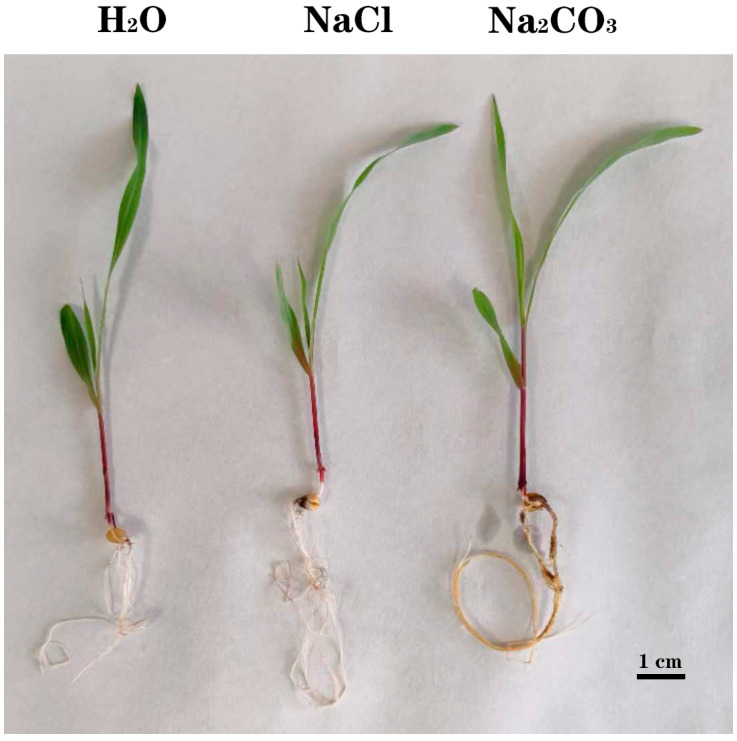
Phenotype of B73 maize roots treated under different conditions for 5 h, scale bar = 1 cm.

B73 maize specimens grown hydroponically in a greenhouse were divided into three groups at the three-leaf stage with uniform growth. One group’s roots were soaked in water for 5 h, while the roots of the other two groups were exposed to 100 mM NaCl and 50 mM Na_2_CO_3_ for the same period. No significant difference was observed between the roots treated with water and NaCl; however, those treated with Na_2_CO_3_ exhibited a notable yellowing (Figure 1). This indicates that maize roots under Na_2_CO_3_ stress may activate distinct metabolic pathways and regulatory networks compared to those treated with water and NaCl.

### 3.2. miRNA Sequencing Results and Data Analysis in Maize Roots under Different Salt Stress Conditions

miRNAs are well known as major post-transcriptional regulators that respond to salt stress in various plants. Roots, being in direct contact with saline soils, are the primary organs affected by salt stress. Therefore, studying the impact of salt stress on maize roots is of great importance.

To identify the differential expression of miRNAs in maize roots under different salt stress conditions, we performed miRNA sequencing analysis on the roots after 5 h of treatment, with three biological replicates for the control (water), NaCl-treated, and Na_2_CO_3_-treated groups. The analysis resulted in 11.91, 12.18, 9.92, 10.54, 14.75, 13.18, 15.61, 13.57, and 19.54 million reads, respectively. The sequences were aligned and filtered against mRNA, RFam (including rRNA, tRNA, snRNA, snoRNA, etc.), and the Repbase database, resulting in the filtered data referred to as ‘Valid data’. The ‘Valid data’ was subsequently analyzed for miRNA identification and prediction (refer to Supplementary Table S1). The majority of the identified miRNAs were 20–24 nucleotides long, representing 95.74% of the total, consistent with the reported length of plant miRNAs (refer to Supplementary Table S2). A total of 325 conserved miRNAs in maize were identified from the miRBase database. In this study, we identified 164 conserved miRNAs and 276 newly predicted miRNAs across 50 families, with 34 families containing multiple miRNA members (refer to Supplementary Tables S3 and S4). The results indicate significant changes in miRNA expression levels in maize roots under various salt stress conditions. Nearly half of the conserved miRNAs showed altered expression, highlighting the extensive impact of salinity and alkalinity on transcriptional regulation in maize.

### 3.3. Differential miRNA Expression Analysis

To investigate the differential expression of miRNAs under different salt stress conditions, particularly carbonate stress, we analyzed miRNA sequencing results. A comparative analysis was performed on miRNA expression levels in the roots of maize subjected to water treatment, 100 mM NaCl treatment, and 50 mM Na_2_CO_3_ treatment. Pearson correlation analysis revealed a strong correlation among the sample groups, confirming the validity of the sampling (refer to Supplementary Figure S1A). The analysis of differentially expressed miRNAs in different comparison groups revealed that three miRNAs showed a differential expression between the NaCl vs. H_2_O group and the Na_2_CO_3_ vs. H_2_O group. There were 29 miRNAs that showed a differential expression between the NaCl vs. H_2_O group and the Na_2_CO_3_ vs. NaCl group. A total of 48 miRNAs showed a differential expression between the Na_2_CO_3_ vs. H_2_O group and the Na_2_CO_3_ vs. NaCl group. Additionally, two miRNAs showed differential expression in all three comparison groups (refer to Supplementary Figure S1B).

**Figure 2:**
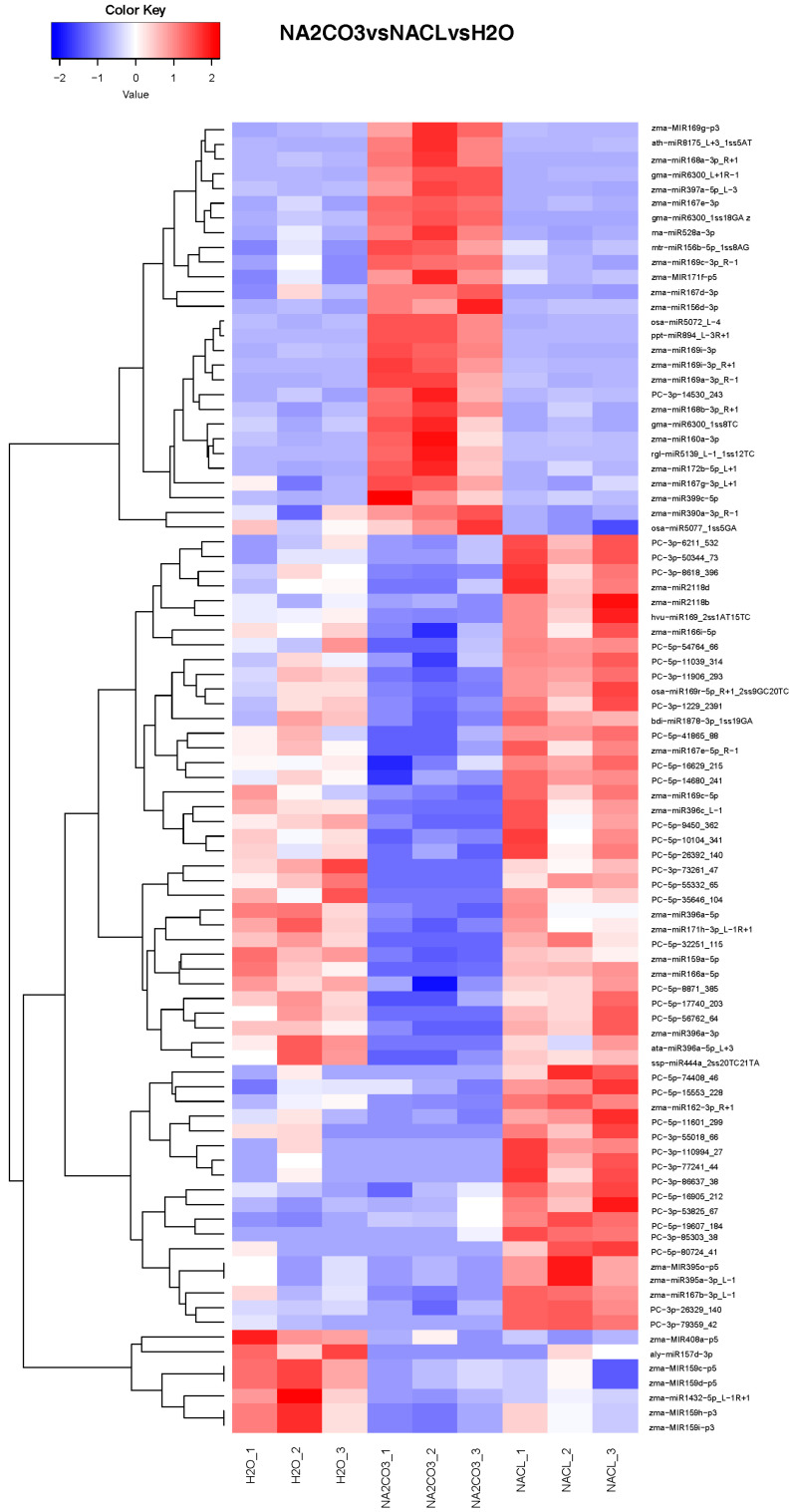
Cluster analysis of differentially expressed miRNAs across various treatment groups.

Significant differences were observed among the groups as follows: in the NaCl vs. H_2_O comparison, forty-one miRNAs exhibited altered expression, with thirty-seven upregulated and four downregulated (refer to Supplementary Table S5); in the Na_2_CO_3_ vs. H_2_O comparison, seventy-four miRNAs showed changes in expression, with twenty-seven upregulated and forty-seven downregulated (refer to Supplementary Table S6); and in the Na_2_CO_3_ vs. NaCl treatment group comparison, one hundred and fifty miRNAs displayed altered expression, with twenty-one upregulated and one hundred and twenty-nine downregulated (refer to Supplementary Table S7). Setting the screening criteria at *p* ≤ 0.05 and a fold change ≥ 2, we identified that in the NaCl vs. H_2_O comparison, 22 miRNAs exhibited altered expression, all of which were upregulated (refer to Supplementary Table S5); in the Na_2_CO_3_ vs. H_2_O comparison, 53 differentially expressed miRNAs were identified, with 22 upregulated and 31 downregulated (refer to Supplementary Table S6). In conclusion, the number of differentially expressed miRNAs in the Na_2_CO_3_ treatment group significantly surpasses that in the NaCl treatment group, suggesting a notably greater impact of carbonate salt on plants compared to neutral salt. Additionally, the clustering analysis of differentially expressed miRNAs reveals notable distinctions between the Na_2_CO_3_ treatment group and the other treatment groups (Figure 2).

### 3.4. Prediction and Bioinformatics Analysis of Differentially Expressed miRNA Target Genes

To explore the regulatory role of miRNAs in maize roots under different salt stress conditions, targeted prediction, GO enrichment, and KEGG pathway analyses were performed on miRNAs uniquely expressed in distinct comparative groups identified through sequencing.

**Figure 3:**
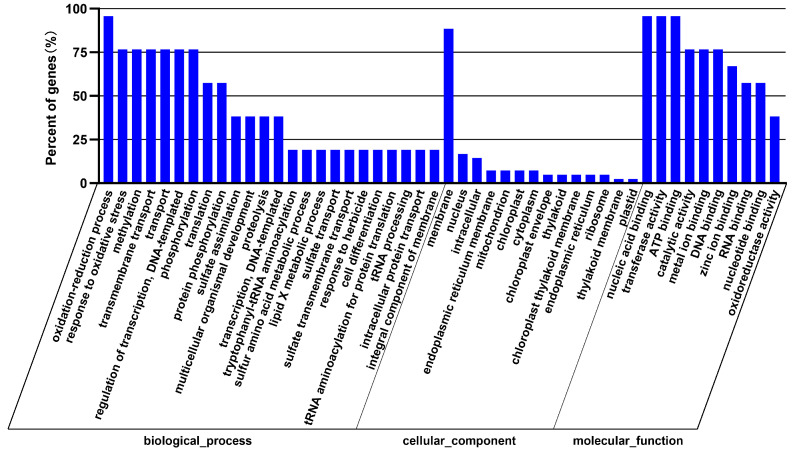
GO functional enrichment analysis of differential miRNA target genes in the NaCl vs. H_2_O comparison group.

**Figure 4:**
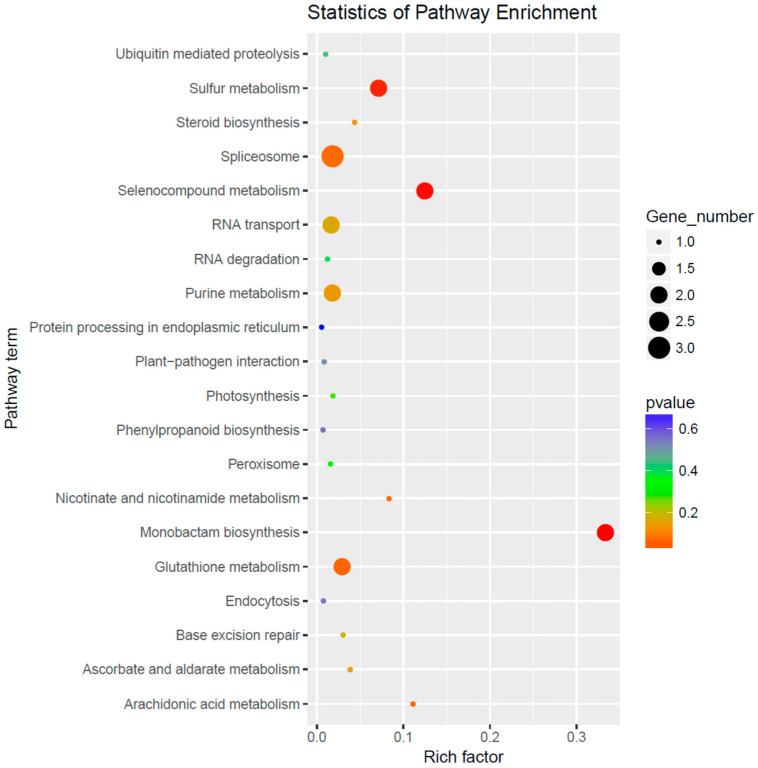
KEGG pathway enrichment analysis of differential miRNA target genes in the NaCl vs. H_2_O comparison group.

The 22 miRNAs exclusively expressed in the NaCl vs. H_2_O group regulate 122 target genes. The GO enrichment analysis indicated that these genes are primarily associated with biological processes such as response to oxidative stress and transmembrane transport, cellular components such as integral components of the membrane, and molecular functions including transferase activity and metal ion binding (Figure 3). Remarkable KEGG pathway enrichment was observed in Monobactam biosynthesis and Selenocompound metabolism, among other pathways (Figure 4). Among these twenty-two differentially expressed miRNAs, two conserved miRNAs, *zma-miR395a-3p_L-1* and *zma-MIR395o-p5* from the MIR395 family, regulate seven genes, including *Zm00001d028164, Zm00001d033981*, and *Zm00001d013296*, which are involved in functions such as Sulfate transporter 2.2, ATP sulfurylase1, and ATP sulfurylase 1 chloroplastic. Their gene functions and enrichment in the GO and KEGG pathways are primarily associated with sulfate synthesis, metabolism, assimilation, and transport pathways, including GO:0000103 (sulfate assimilation), GO:0008272 (sulfate transport), and ko00920 (Sulfur metabolism). Additionally, other non-conserved miRNAs regulate genes associated with sulfate metabolism. For instance, *Zm00001d040796*, regulated by *PC-5p-19607_184* and annotated as Ubiquitin-like-specific protease ESD4, is enriched in GO:0008234 (cysteine-type peptidase activity), and *Zm00001d028818*, regulated by *PC-3p-45471_81* and annotated as Calpain-type cysteine protease DEK1, is enriched in GO:0008234 (cysteine-type peptidase activity) (refer to Supplementary Table S8). These findings indicate that NaCl treatment influences sulfate metabolism in maize roots.

**Figure 5:**
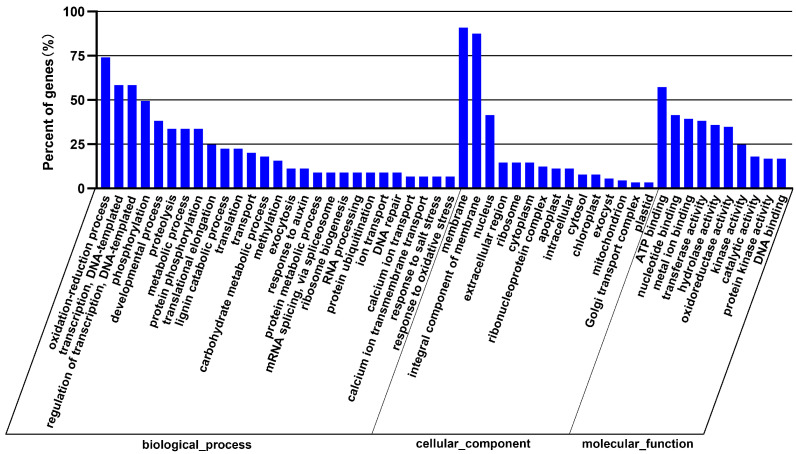
GO functional enrichment analysis of differentially expressed miRNA target genes in the Na_2_CO_3_ vs. H_2_O comparison group.

**Figure 6:**
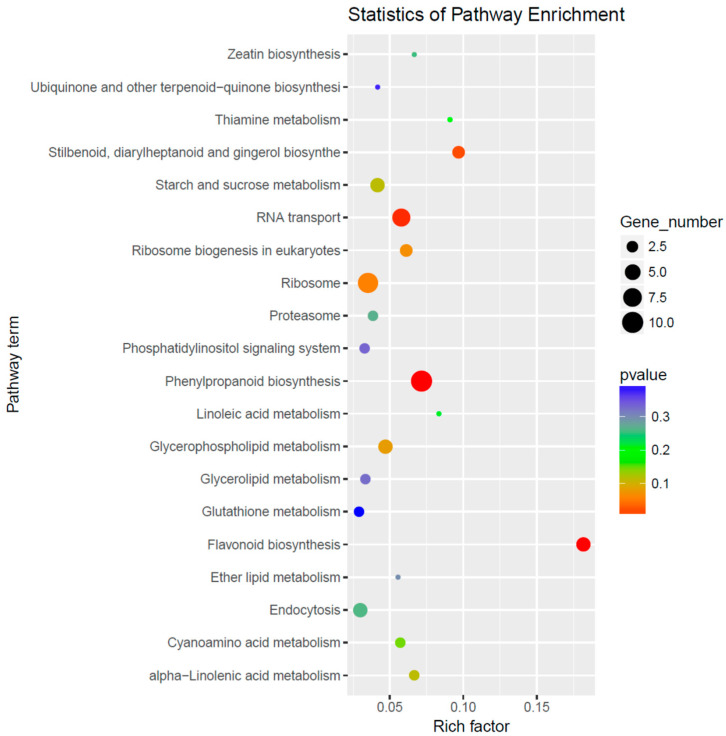
KEGG pathway enrichment analysis of differentially expressed miRNA target genes in the Na_2_CO_3_ vs. H_2_O comparison group.

In the Na_2_CO_3_ vs. H_2_O comparison group, 53 uniquely expressed miRNAs regulate 379 target genes. The GO enrichment analysis reveals that these genes are primarily associated with biological processes such as the oxidation–reduction process, exocytosis, heme biosynthetic process, calcium ion transport, and lignin catabolic process; cellular components such as the extracellular region and exocyst; and molecular functions including copper ion binding and oxidoreductase activity (Figure 5). A remarkable KEGG pathway enrichment is observed in Phenylpropanoid biosynthesis, Flavonoid biosynthesis, Stilbenoid, diarylheptanoid, and gingerol biosynthesis pathways (Figure 6). Out of these 53 differentially expressed miRNAs, 32 are conserved miRNAs, belonging to the MIR156, MIR159, MIR167, MIR168, MIR169, MIR171, MIR396, MIR397, and MIR528 families. The 238 genes they regulate are involved in various functions, including *Zm00001d030062,* regulated by *zma-miR156d-3p* and annotated as LRR protein 4; *Zm00001d029550*, regulated by *zma-miR159a-5p* and annotated as Diacylglycerol kinase 1; *Zm00001d024744*, regulated by *zma-miR167g-3p_L+1* and annotated as PTI1-like tyrosine-protein kinase; *Zm00001d047683*, regulated by *zma-miR168b-3p_R+1* and annotated as NAD(P)-binding Rossmann-fold superfamily protein; *Zm00001d007181*, regulated by *zma-miR169i-3p* and annotated as Calcium-binding protein; *Zm00001d041592*, regulated by *zma-MIR171f-p5* and annotated as modifier of snc1; *Zm00001d033876*, regulated by *zma-miR396a-5p* and annotated as Growth-regulating factor 2; *Zm00001d017762*, regulated by *zma-miR397a-5p_L-3* and annotated as abscisic acid 8’-hydroxylase1; *Zm00001d020764*, regulated by *zma-miR528a-3p* and annotated as carbonic anhydrase5; and *Zm00001d020764*, regulated by *zma-miR528a-3p* and annotated as carbonic anhydrase5 (Supplementary Table S9).

### 3.5. Analysis of Differentially Expressed miRNAs Shared by Different Comparison Groups

To gain further insights into the potential mechanisms of miRNA responses to different salt stress conditions in maize roots, we analyzed the overlapping differentially expressed miRNAs between the NaCl vs. H_2_O and Na_2_CO_3_ vs. H_2_O comparison groups. We identified 36 differentially expressed miRNAs exhibiting consistent expression patterns in both groups, with 21 upregulated and 15 downregulated miRNAs (Supplementary Table S10). The upregulated conserved miRNAs belong to the MIR395, MIR172, MIR171, MIR169, MIR168, MIR162, and MIR156 families. The target genes of these miRNAs, including *Zm00001d040583*, *Zm00001d024477*, *Zm00001d009858*, *Zm00001d034635*, *Zm00001d039971*, *Zm00001d036593*, *Zm00001d014568*, etc., are enriched in the GO pathways, with processes such as metal ion binding, iron ion binding, flavonoid biosynthetic process, response to hormone, cellular response to DNA damage stimulus, DNA repair, and auxin-activated signaling pathway. In contrast, the downregulated, conserved miRNAs are primarily distributed among the MIR408, MIR396, MIR171, and MIR159 families. The target genes of these miRNAs, including *Zm00001d051265*, *Zm00001d019670*, *Zm00001d038107*, *Zm00001d007638*, etc., are enriched in the GO pathways via factors such as the integral component of the membrane, the regulation of the gibberellic acid-mediated signaling pathway, and the lipid metabolic process.

Apart from the miRNAs with consistent expression patterns, a subset of differentially expressed miRNAs showed contrasting expression patterns between the comparison groups (refer to Supplementary Table S11). A total of 58 miRNAs were found to be upregulated in the NaCl vs. H_2_O group but downregulated in the Na_2_CO_3_ vs. H_2_O group. These predominantly conserved miRNAs are members of the MIR162, MIR166, MIR167, MIR168, MIR169, MIR395, and MIR396 families. The target genes of these miRNAs, such as *Zm00001d018977*, *Zm00001d027412*, *Zm00001d018977*, *Zm00001d034493*, *Zm00001d026590*, *Zm00001d027874*, *Zm00001d028164*, *Zm00001d033981*, *Zm00001d015410*, etc., are enriched in GO pathways via factors including DNA binding, ATP binding, integral component of membrane, riboflavin biosynthetic process, response to hormone, CCAAT-binding factor complex, sulfate assimilation, metal ion binding, and more.

Conversely, 11 miRNAs were found to be downregulated in the NaCl vs. H_2_O group but upregulated in the Na_2_CO_3_ vs. H_2_O group. These predominantly conserved miRNAs belong to the MIR156, MIR167, MIR168, MIR169, MIR397, MIR528, and MIR2118 families. Their target genes, such as *Zm00001d004553*, *Zm00001d047587*, *Zm00001d024744*, *Zm00001d028837*, *Zm00001d006682*, *Zm00001d015592*, *Zm00001d024477*, *Zm00001d033855*, *Zm00001d009532*, *Zm00001d020764*, etc., are associated with GO pathways via factors such as metal ion binding, glucose-6-phosphate dehydrogenase activity, peptidyl-tyrosine phosphorylation, nucleic acid binding, anaphase-promoting complex, oxidation-reduction process, integral component of membrane, auxin-activated signaling pathway, carbonate dehydratase activity, and more.

### 3.6. qPCR Validation of Sequencing Results

We selected 10 miRNAs from Supplementary Tables S10 and S11 for qPCR validation experiments. The qPCR validation results matched the sequencing data, confirming the reliability of the sequencing results (refer to Supplementary Figure S2).

### 3.7. The Changes in the Content of Different Ions in Maize Roots under Neutral and Alkaline Salt Stress

**Figure 7:**
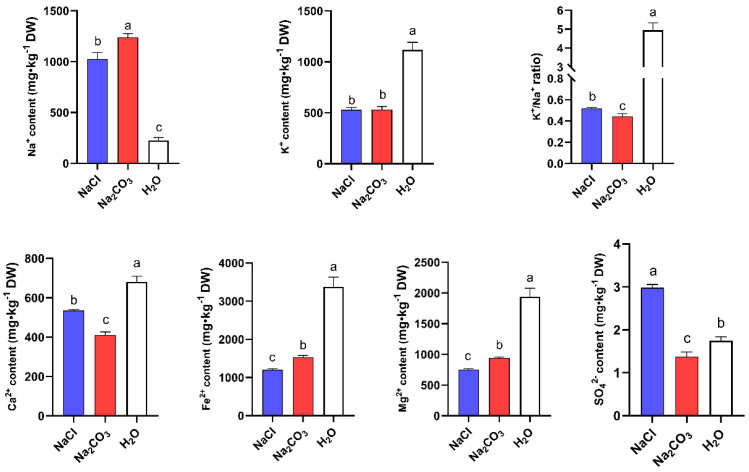
Alterations in ion content in maize roots under neutral and alkaline salts stresses, with different lowercase letters indicating statistical significance.

Excessive salt in the soil inhibits the absorption, translocation, and distribution of nutrients in plants, leading to an imbalance in plant ionic composition, thereby affecting plant physiological traits. Therefore, combining phenotypic observations and data analysis, we aim to investigate the changes in physiological indicators for each group under various salinity and alkalinity stress conditions, particularly the alterations in cations and anions, which could offer theoretical insights into the internal regulatory network shifts in maize under different salt–alkaline stresses. The testing of physiological and biochemical indicators in roots such as sodium, potassium, sulfate, calcium, and iron ions is proposed. We found that the content of various ions in different treatment groups was different. Compared with the normal water treatment group, except for Na^+^ and SO_4_^2−^, the content of other ions showed a downward trend in the NaCl and Na_2_CO_3_ treatment groups. However, the degree of reduction was different, with Na^+^, Ca^2+^, SO_4_^2−^, and the K^+^/Na^+^ ratio being higher in the NaCl treatment group than in the Na_2_CO_3_ treatment group, except for K^+^ content. In contrast, Fe^2+^ and Mg^2+^ showed an opposite trend, with these two metal ions having higher content in the Na_2_CO_3_ treatment group. The content of SO_4_^2−^ showed an upward trend in the NaCl treatment group and a downward trend in the Na_2_CO_3_ treatment group (Figure 7). These results suggest that plants mobilize different metabolic networks in response to neutral and alkaline salt stress.

## 4. Discussion

Differential expression patterns of the same miRNA between salt-sensitive and salt-tolerant plants under salt stress indicate that the changes in miRNA expression may serve as a response mechanism to varying salt concentrations. Zhou et al. [24] employed a quantitative real-time polymerase chain reaction (qRT-PCR) to select five target genes belonging to the miRNA family, which are known to play a role in plant responses to salt stress. The study of plant miRNAs gained significant momentum from 2002 onwards, coinciding with the discovery of plant proteins involved in miRNA biogenesis. Satendra K et al. [25] provided a concise historical account highlighting the importance of miRNA discovery.

To identify the potential interactions between miRNAs and mRNAs in response to salt stress, a co-expression analysis was conducted using the same samples. The comparative expression analysis during germination, under both threshold (273 mM) and optimal (43 mM) NaCl treatments, revealed 13 differentially expressed miRNAs along with 23 corresponding target mRNAs. Furthermore, a comparison between forty-three mM NaCl and non-salt-stress conditions identified one differentially expressed miRNA and its corresponding target mRNA [26]. These findings furnish fundamental data for further investigation into the molecular mechanisms involved in the germination of salt-stressed seeds, while also serving as a reference for advancing salt tolerance during plant germination. In this study, we analyzed differentially expressed miRNAs in corn under NaCl stress and also under alkaline salt Na_2_CO_3_ stress, identifying 41 and 74 different miRNAs under these two stress conditions, respectively. We also found that under NaCl stress, a larger number of differentially expressed miRNAs were upregulated, accounting for over 90% of the total, whereas only 36% of the miRNAs were upregulated under Na_2_CO_3_ stress. Analyzing the number and expression patterns of different miRNAs in the two comparison groups also demonstrates that the factors mobilized by plants in response to Na_2_CO_3_ stress are much greater than those to NaCl stress, involving a more complex metabolic regulatory network mechanism.

In this study, we found that members of the MIR156, MIR169, MIR168, MIR162, MIR395, and MIR396 families were enriched in different treatment groups, with both similar and different expression patterns. This suggests that these miRNA members from the families may be involved in plant responses to different salt–alkali treatments, but their metabolic regulatory networks and mechanisms of action may be more complex. Wan et al. [27] found that the Osa-miR168-OsAGO1 module is involved in regulating rice salt stress tolerance. Researchers used STTM technology to silence miR168, which enhanced rice salt stress tolerance, with mutant plants showing significantly more roots and longer roots and stems under salt stress compared to wild-type plants. In this study, *zma-miR168a-5p* was downregulated under NaCl stress and upregulated under Na_2_CO_3_ stress. Its target gene, *Zm00001d006682*, is indeterminate domain 8, a member of the IDD family. IDD family genes encode transcription factors with zinc finger proteins and play a broad role in plant growth and development [28,29,30,31]. In Arabidopsis, Welch et al. [32] found that IDD transcription factor members JKD and MGP are part of the SCR-SHR complex. JKD and MGP are specifically expressed in root stem cells and are regulated by the *SCR* and *SHR* genes. Additionally, *SGR5* is expressed in the endodermis of the inflorescence stem, participating in the early gravitropic response of Arabidopsis stems [33]. It is evident that IDD genes not only regulate plant flowering but also participate in the regulation of plant growth and development. *zma-miR396c_L-1* was upregulated under NaCl stress and downregulated under Na_2_CO_3_ stress, regulating the target gene *Zm00001d018260*, which is Growth-regulating factor 6 (*GRF6*). At the transcriptional level, GRFs are typically regulated by the microRNA miR396 [34], and some studies have revealed other functions of GRFs in plant biology, such as flowering, seed, and root development; growth control under stress conditions; and the regulation of plant lifespan [35,36,37,38,39,40,41]. Yuan et al. [42] found that the Osa-miR396c-GRF module plays a significant role in regulating the salt stress tolerance of creeping bentgrass. The overexpression of *Osa-miR396c* in creeping bentgrass resulted in significantly reduced tiller number and length, and narrower and shorter leaves. Under salt stress, mutant plants showed increased leaf water content and chlorophyll content, reduced electrolyte leakage, and notably, an elevated expression of SALT OVERLY SENSITIVE1 (*OsSOS1*), leading to a decrease in the relative Na^+^ content and an increase in the K^+^/Na^+^ ratio, demonstrating enhanced salt tolerance.

Members of the MIR408, MIR159, MIR171, and MIR172 families exhibited the same expression patterns in the two comparison groups, suggesting that these miRNA members may play a necessary role in plant responses to different salt–alkali treatments. In this study, *zma-miR159a-5p* was found to be downregulated in both comparison groups, regulating the target gene *Zm00001d029550*, which is diacylglycerol kinase 4. Diacylglycerol kinase (DGK) catalyzes the phosphorylation of lipids to produce diacylglycerol phosphate (PA), playing a significant role in biological activities. Previous studies have found that DGK plays an important role in plant responses to stress, such as in Arabidopsis, where Li et al. [43] discovered that *DGK5* and its catalytic product PA bind to *ABA2* and inhibit its activity, thereby affecting ABA synthesis under stress conditions and the plant’s ability to cope with stress. Another differentially expressed miRNA, *zma-miR172e_L-1*, was upregulated in both comparison groups, regulating the target gene *Zm00001d035512*, which is AP2-EREBP-transcription factor 81. miR172, a highly conserved member of the miRNA family, is induced by various stresses such as low temperature, salt, and drought, and can also participate in plant responses to various environmental stresses by regulating AP2-type transcription factors [44,45,46,47]. Overexpressing *miRNA172c* or knocking out GmNNC1 in soybean can enhance its salt tolerance; conversely, interfering with *miR172* expression or overexpressing *GmNNC1* increases soybean’s salt sensitivity [48]. Additionally, overexpressing soybean *miR172c* in Arabidopsis can significantly enhance its tolerance to drought and salt stress [49]. Although there is a relatively clear understanding of the mechanisms by which miR172 mediates plant responses to stress in Arabidopsis and soybean, research on other plants, especially important crops like corn, remains at the level of genome-wide expression analysis and has not yet involved the analysis of specific molecular mechanisms and the construction of regulatory networks.

Furthermore, members of the MIR166, MIR167, MIR397, MIR528, and MIR2118 families exhibited opposite expression patterns in the two comparison groups, indicating that these family members played unique roles under different salt and alkali stresses. *zma-miR167a-5p* was upregulated under NaCl stress and downregulated under Na_2_CO_3_ stress, regulating the target gene *Zm00001d026590*, which is ARF-transcription factor 30. Liu et al. [50] discovered that Zma-miR167 could reduce the transcription of *ZmARF3* and *ZmARF30*, resulting in decreased transcription levels of *ZmPAO1* and producing less hydrogen peroxide (H_2_O_2_), thereby enhancing maize’s resistance to maize chlorotic mottle virus (MCMV). *zma-miR528a-3p* was downregulated under NaCl stress and upregulated under Na_2_CO_3_ stress, regulating the target gene *Zm00001d020764*, which is carbonic anhydrase5. Carbonic anhydrases (CAs) are a class of zinc-containing metalloenzymes that play an important role in various physiological processes such as cellular pH regulation, carbon dioxide transport, electrolyte balance, and cell homeostasis, which are crucial for cell survival and proliferation. The carbonyl sulfide (COS) absorbed by plants can be converted into CO_2_ and Hydrogen Sulfide (H_2_S) by carbonic anhydrase (CA) in cells [51]. High concentrations of H_2_S tend to bind with ferrous ions, thereby modifying the structure of proteins containing ferrous ions (such as cytochrome oxidase, hemoglobin, myoglobin, etc.), which inhibits their activity and function, thus exhibiting cytotoxic effects [52,53,54,55].

In summary, miRNAs play a crucial role in plants’ response to alkaline salt stress, but the current research on this topic is not sufficient. The specific effects of alkaline salt stress on plant miRNA expression and its regulatory mechanisms require further in-depth investigation. Our research aims to provide a theoretical basis for comprehensively understanding how crops respond to complex alkaline salt stress and for improving saline–alkali soils to expand crop cultivation areas. Through this research, we hope to provide a scientific basis for agricultural production, helping farmers to increase crop yields under saline–alkali conditions.

## 5. Conclusions

This study reveals the impact of soil salinization, a complex environmental factor, on plant gene expression by analyzing the differential expression of miRNAs in the roots of maize seedlings after treatment with 100 mM NaCl, 50 mM Na_2_CO_3_, and H_2_O for 5 h. The results show that the Na_2_CO_3_ treatment group exhibited the highest number of differentially expressed miRNAs. The cluster analysis indicates that these differentially expressed miRNAs are primarily involved in the regulation of ion transport, binding, metabolism, phenylpropanoid, and flavonoid biosynthesis pathways. In contrast, the unique, differentially expressed miRNAs in the NaCl treatment group are associated with the sulfur metabolism pathways. This suggests that there are significant differences in the response patterns of maize to different saline–alkaline treatments. This study provides a theoretical basis and genetic resources for further analysis of the molecular mechanisms underlying salt–alkali tolerance in maize.

## Data Availability

All relevant data are provided as figures or table within the paper.

## Supplementary Materials

The following supporting information can be downloaded at: https://www.mdpi.com/article/10.3390/cimb46080524/s1.
