# Supplementary material for: miRNA Sequencing Analysis in Maize Roots Treated with Neutral and Alkaline Salts
Source: Curr Issues Mol Biol. 2024 Aug 15;46(8):8874–89. doi: 10.3390/cimb46080524 (PMC11352498; doi:10.3390/cimb46080524)
Supplement: Supplementary file 1 [file cimb-46-00524-s001.zip › cimb-3112744-supplementary.pdf]

Supplementary Figures

A

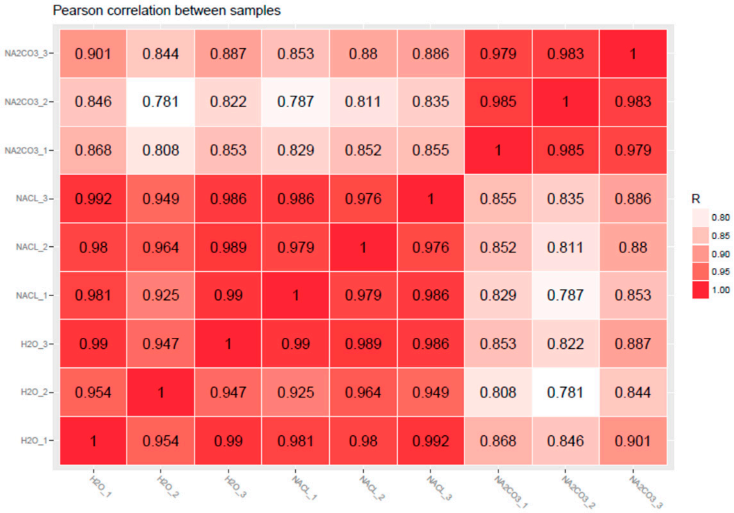

B

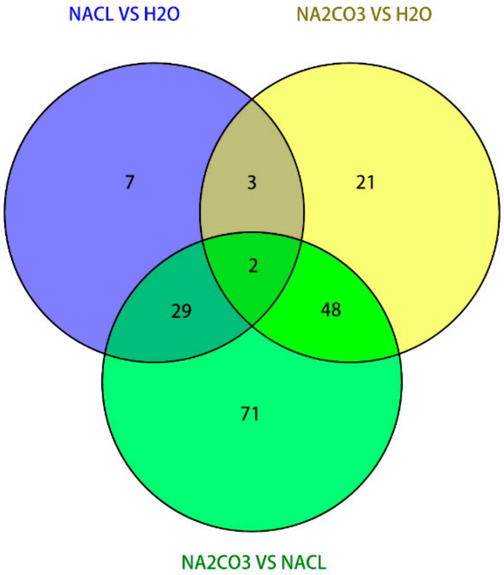

Supplementary Figure 1.

(A) Pearson correlation analysis among samples across different treatment groups; a darker color and values closer to 1 indicate higher correlation.

(B) Venn diagram of differentially expressed miRNAs in different comparison groups.

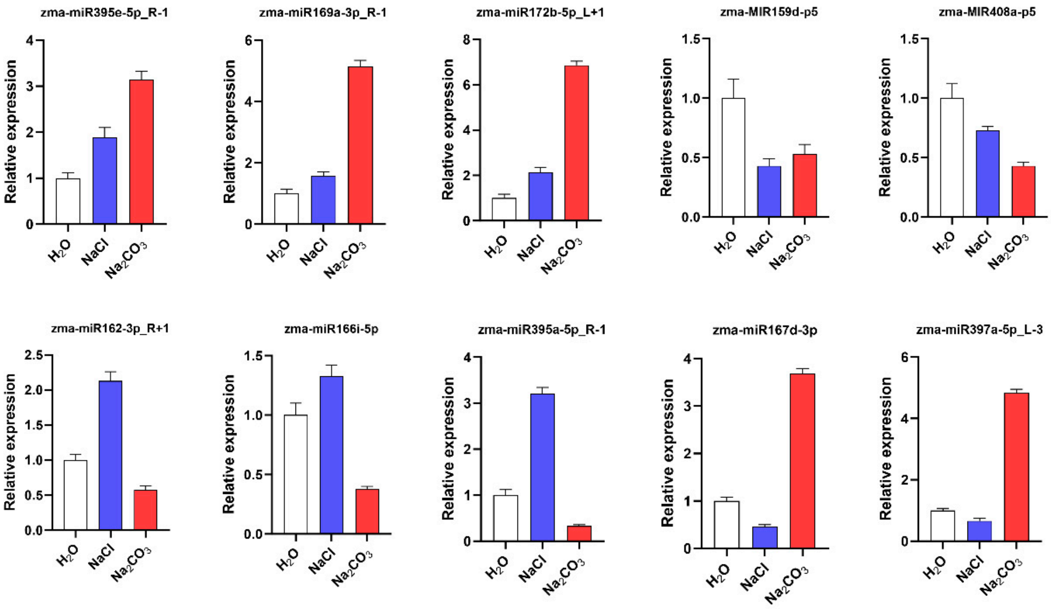

Supplementary Figure 2. qPCR validation of the expression patterns of 10 selected miRNAs under Neutral and Alkaline Salts stresses.
